# Developing an innovative national ACP-OSCE program in Taiwan: a mixed method study

**DOI:** 10.1186/s12909-024-05294-5

**Published:** 2024-03-23

**Authors:** Yen-Lin Wu, Tsu-Yi Hsieh, Sheau-Feng Hwang, Yi-Yin Lin, Wei-Min Chu

**Affiliations:** 1 Department of Emergency Medicine, Taichung Veterans General Hospital, Taichung, Taiwan; 2 Division of Clinical Training, Department of Medical Education, Taichung Veterans General Hospital, Taichung, Taiwan; 3 Department of Allergy-Immunology-Rheumatology, Taichung Veterans General Hospital, Taichung, Taiwan; 4 Department of Obstetrics, Gynecology and Women’s Health, Taichung Veterans General Hospital, Taichung, Taiwan; 5 Hospice Foundation of Taiwan, Taipei, Taiwan; 6 Department of Family Medicine, Taichung Veterans General Hospital, Taichung, Taiwan; 7 School of Medicine, National Yang Ming Chiao Tung University, Taipei, Taiwan; 8 Department of Post-Baccalaureate Medicine, College of Medicine, National Chung Hsing University, Taichung, Taiwan; 9 Department of Epigemiology on Aging, National Center for Geriatrics and Gerontology, Obu, Japan; 10 Geriatrics and Gerontology Research Center, College of Medicine, National Chung Hsing University, Taichung, Taiwan

**Keywords:** Advance care planning, Objective structured clinical examination, Medical education, Communication, Mix-method study

## Abstract

**Objectives:**

To evaluate the process and the comprehensiveness of advance care planning (ACP), we designed a national ACP-OSCE (Objective Structured Clinical Examination) program.

**Methods:**

The program was designed as a 40-minute OSCE test. Participants were categorized as different ACP team members to illustrate realistic scenarios. Preceptors were asked to observe ACP professionals’ actions, responses, and communication skills during ACP with standardized patients (SP) through a one-way mirror. Participants’ communication skills, medical expertise, legal knowledge, empathetic response and problem-solving skills of ACP were also self-evaluated before and after OSCE. Thematic analysis was used for qualitative analysis.

**Results:**

In Nov 2019, a total of 18 ACP teams with 38 ACP professionals completed the ACP-OSCE program, including 15 physicians, 15 nurses, 5 social workers, and 3 psychologists. After the ACP-OSCE program, the average score of communication skills, medical expertise, legal knowledge, empathetic response, ACP problem-solving all increased. Nurses felt improved in medical expertise, legal knowledge, and problem-solving skills, psychologists and social workers felt improved in legal knowledge, while physicians felt no improved in all domain, statistically. Thematic analysis showed professional skills, doctoral-patient communication, benefit and difficulties of ACP were the topics which participants care about. Meanwhile, most participants agreed that ACP-OSCE program is an appropriate educational tool.

**Conclusion:**

This is the first national ACP-OSCE program in Asia. We believe that this ACP-OSCE program could be applied in other countries to improve the ACP process and quality.

**Supplementary Information:**

The online version contains supplementary material available at 10.1186/s12909-024-05294-5.

## Backgrounds

Since the 90s, patients’ awareness of issues related to making their own treatment-related decisions during the end of life has increased [1–3]. However, some patients are unable to make such decisions because of consciousness disturbance or delirium. Thus, advance care planning(ACP) plays an important role in meeting that need. ACP is a process for patients to make decisions about his or her future health care in consultation with health care providers, family members, and important others [4]. Initially, the purpose of ACP was to complete paper documents, such as advance decision (AD), Do-Not-Resuscitate (DNR) order, and Do-Not-Hospitalise (DNH). However, the spirit of ACP today involves not only paper documents, but also the discussion process and the underlying meaning [5]. The first official approval of advance directive was in 1976. It was believed that patients who had lost the capacity to make a decision could receive the treatment that they had hoped for if they had appointed a Health Care Agent (HCA) or had documented their decisions in advance. In 1990, the United States Congress legislated the “Patient Self-Determination Act”, and asked all medical insurance certified medical facilities to provide information about AD for patients [6]. More and more research has shown that ACP could improve the quality of life for patients with incurable diseases, especially for the elderly living in nursing facilities or at the end of life [7–12].

During implementation of ACP, some obstacles related to medical professionals were discovered. Some ACP professionals had difficulties with communication skills or counseling skills [13]. These difficulties may arise from uncertainty of prognosis, fear of introducing a painful memory, or unpreparedness for upcoming conversations [14]. Some difficulties during ACP were related to lack of medical knowledge [15, 16], while others were related to empathetic responses when dealing with emotions [17]. Some difficulties were related to insufficient legal knowledge [18]. Some patients even go to lawyers for ACP because they believe that lawyers can better understand their socio-economic background and make proper plans accordingly [19]. However, other research shows that through properly designed workshops, role-play, and group discussions, these obstacles can be managed [15].

Communication skills can be taught and trained, especially using deliberate practice such as Objective Structured Clinical Examination (OSCE) [20–22]. The OSCE was first proposed by Harden in 1975, as a tool for standardized evaluation of medical students’ performance during work [23]. The purpose of OSCE is to evaluate clinical skills and knowledge in a standardized clinical setting using a designed checklist with a reproducible method to ensure high quality. Standardized patients (SP) present with multiple illnesses so that participants can undergo a standardized evaluation [24]. OSCE has proven to be an effective evaluation method for medical students and residents in various medical fields, such as geriatrics [25], surgery [26] and radiology [27].

For ACP, adequate training can improve readiness among healthcare professionals [28]. However, there was no standardized method for education and evaluation for ACP professionals. In Taiwan, ACP education is categorized as continuous medical education rather than pre-graduate education. According to the guidelines outlined by the Ministry of Health and Welfare in Taiwan, titled “Regulations for the Recognition of Qualifications and Conduct of Courses for Advance Care Planning (ACP) Counselors,” individuals, including physicians, nursing professionals, or social workers, who have completed a sufficient number of hours in either online or on-site courses, are eligible to become qualified ACP professionals. In January 2019, Taiwan officially approved the Patient Right to Autonomy Act which allows all citizens in Taiwan to make AD for 5 critical conditions. According to the law, all citizens wishing to make an AD must go through official ACP performed by certified healthcare professionals (HCP). This study aimed to investigate the outcome after implementing a designed OSCE program in ACP for medical professionals in Taiwan.

## Methods

### Data source

This study aimed to evaluate the effect of the ACP-OSCE program on ACP skills among ACP healthcare professionals. In 2019, there were 20 hospitals which were designated as ACP demonstration hospitals by Taiwan’s Ministry of Health and Welfare, in every city nationwide. The Hospice Foundation was responsible for the education and evaluation of the demonstration hospitals. Due to the absence of relevant literature for the ACP-OSCE, a pilot study was conducted prior to the main analysis, involving the collection of data from 20 cases. This preliminary investigation aimed to estimate the required sample size. Results for the Communication Skills group revealed an average score of 3.55 with a standard deviation of 0.686 before the OSCE, and an average score of 3.85 with a standard deviation of 0.587 after the OSCE. Using Gpower software for sample size estimation, it was determined that a sample size of 38 would be necessary to achieve a statistical power of 0.8. Thus, on November 16, 2019, national ACP-OSCE was held for ACP healthcare professionals from demonstration hospitals. We enrolled 45 ACP healthcare professionals from 20 hospitals, including physicians, nurses, psychologists, and social workers in the final evaluation.

### ACP-OSCE program structure and evaluation criteria

The ACP-OSCE consists of a 40-minute test and a 20-minute feedback session. The scenarios and checklists for ACP-OSCE were developed by the Hospice Foundation of Taiwan, which engaged local experts in palliative care and medical ethics as consultants forming a dedicated working group. This working group, through consensus-building and discussions, crafted relevant scenarios and assessment checklists. Subsequently, these materials underwent expert validation. The main purpose of this program was to evaluate how ACP healthcare professionals answer questions from participants and explain the rights and obligations of the “Patient Right to Autonomy Act”, the choices of AD, the differences of each special clinical conditions, the rights of the HCA, and future problems when executing AD. We also wanted to evaluate ACP professionals’ skills in dealing with emotions and special events during ACP. The scenario designed was a common situation that ACP professionals often deal with. Before the exam, we grouped all ACP healthcare professionals according to their hospitals and medical professions. Each team consisted of one physician, and at least one other professional such as a nurse, social worker, or psychologist.

The checklists for ACP-OSCE constitute a semi-structured questionnaire. It comprises both open-ended and closed-ended questions, including 15 items for participants to assess their communication skills, medical expertise, legal knowledge, empathetic response, and problem-solving skills. Additionally, there are open-ended comments soliciting participants’ experiences, perspectives on what they have learned, and the most memorable aspects. Before and after the examination, identical questionnaires and satisfaction queries were distributed to all participants for survey purposes. The responsible instructors provided explicit instructions, including ensuring anonymous submission methods and assuring participants that their feedback would not impact their grades.

### Taiwan’s ACP training mandates: exploring course structure and topics

In Taiwan, according to the “Patient Right to Autonomy Act”, physicians, nurses, psychologists, and social workers are required to complete a 4-hour, 6-hour, and 11-hour course on ACP, respectively. The course topics include “Patient Right to Autonomy Act introduction and related laws”, “AD and palliative care”, “ACP process and communication skill”. We also documented ACP experience in number of cases for each participants. We did not collect the years of experience for each participant, because ACP is a relatively new learning experience for a majority of healthcare professionals in Taiwan. Given this context, we believe that using the number of ACP cases can better represent relevant experience, as the Patient Right to Autonomy Act is a recent development. By utilizing the number of ACP cases, we aim to account for and enhance the representation of experience across different professional roles, thereby augmenting the average number of ACP cases conducted within each professional category.

### ACP-OSCE implementation: role of standardized patients and preceptors

Forty-five trained SPs were recruited for the ACP-OSCE. SPs were provided with “guidance of SP” with instructions about roles with specific lines in the scenario to minimize differences in performance. After the exam, in addition to providing all participants with the same questionnaire as well as questions about satisfaction, we provided open-ended feedback.

Nine experienced physicians and nurses were recruited as Preceptors. The Hospice Foundation of Taiwan, in the development of the ACP-OSCE, engaged local experts in palliative care and medical ethics as consultants, establishing a working group. All members of this group are healthcare professionals with over 10 years of clinical and academic experience in palliative care. Prior to the implementation of the ACP-OSCE program, a pre-program expert consensus meeting was conducted to mitigate potential inter-rater differences and ensure a standardized approach. Preceptors were provided with “guidance for preceptors” consisting of standardized grading instructions in advance in order to minimize inter-rater differences, and were then required to observe the whole ACP process behind a one-way mirror. We also provided preceptors with a checklist to evaluate each ACP team’s performance related to communication skills, medical expertise, legal knowledge, empathetic response, and problem-solving skills during ACP. Supplementary Data 1 provides a detailed description of our ACP scenario, as well as the instructions for participants, preceptors, and SPs.

### Statistical analyses

We applied mixed-methods evaluation approach to analyze our data. Qualitative information was analyzed using thematic analysis [29, 30] and a general inductive approach described below [31]: the five longest responses were selected and read by two researchers with training in medical professionalism to find meaningful words and phrases related to the purpose of this research for preliminary coding. Next, the researchers compared, discussed, and integrated each other’s codes to form an initial classification. If there was a disagreement, a third researcher reviewed it. The process was repeated until consensus was reached, and no new codes emerged. Qualitative data was translated from Chinese to English and proofread by native English speaker to ensure the precision of the original text. Paired t test was used for analysis of the differences in self-confidence in communication skills, medical expertise, legal knowledge, empathetic response, and problem-solving skills before and after ACP-OSCE. A two-tailed p value < 0.05 was considered statistically significant. Statistical analyses were performed using SAS version 9.4 (Statistical Analysis Software 9.4, SAS Institute Inc., Cary, North Carolina, USA).

### Patient and public involvement

Patients or the public were not involved in the design or conduct of the study. We will continue to disseminate the results of the study to the public through various media channels by the Hospice Foundation of Taiwan.

## Results

Forty-five ACP healthcare professionals were enrolled and given questionnaires before and after the exam. Thirty-eight questionnaires were retrieved, with a response rate of 84%. Among the 38 participants, there were 15 physicians, 15 nurses, 3 psychologists, and 5 social workers. The basic characteristics of the 38 ACP professionals are shown in Table 1. Table 2 shows the average score of communication skills, medical expertise, legal knowledge, empathetic response, and problem-solving skills before and after the exam among all participants. There was a significant increase in communication skills, medical expertise, legal knowledge, and problem-solving skills.

Table 3 shows the subgroup analysis of physicians, nurses, psychologists, and social workers. For physicians, the average score of the 5 domains of ACP skills increased without significance. For nurses, the average score of medical expertise, legal knowledge, and consultation problem solving skills were significantly increased. As for psychologists and social workers, only legal knowledge was significantly increased.

**Fig. 1:**
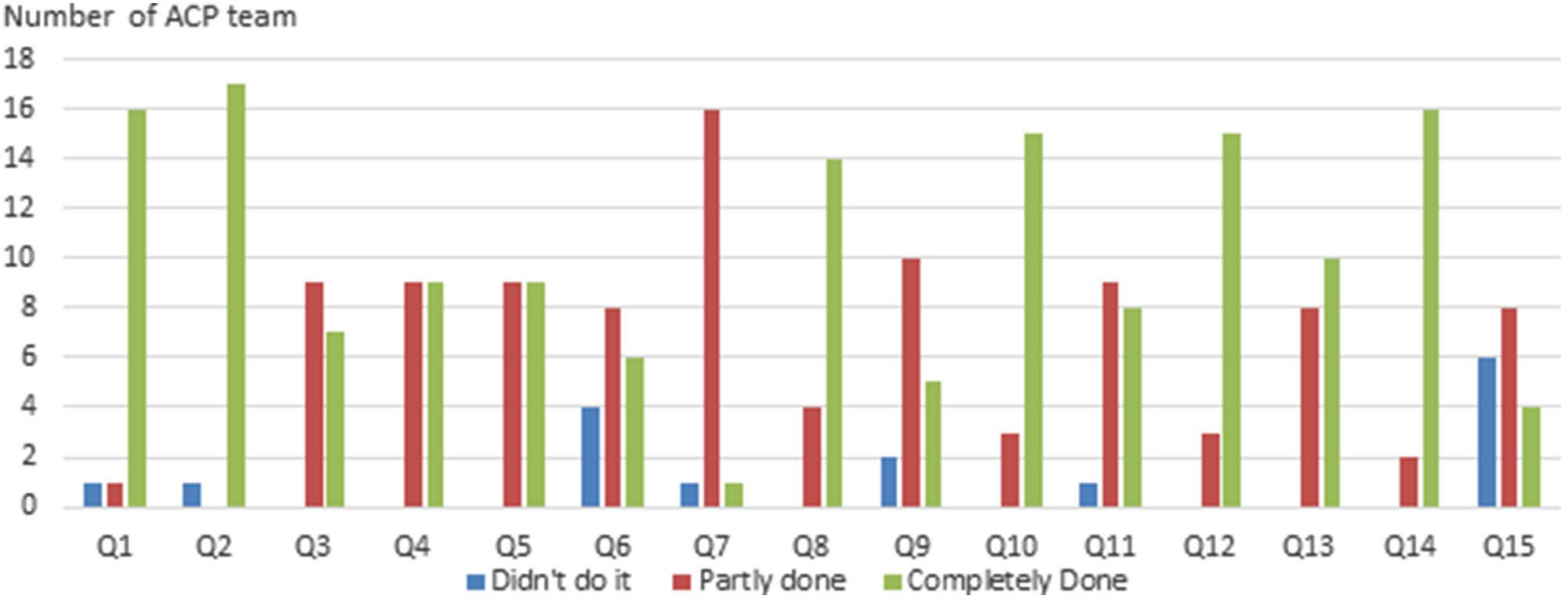
Score reflecting the 15 domains of the ACP performed by preceptors in their evaluation of each ACP team

**Fig. 2:**
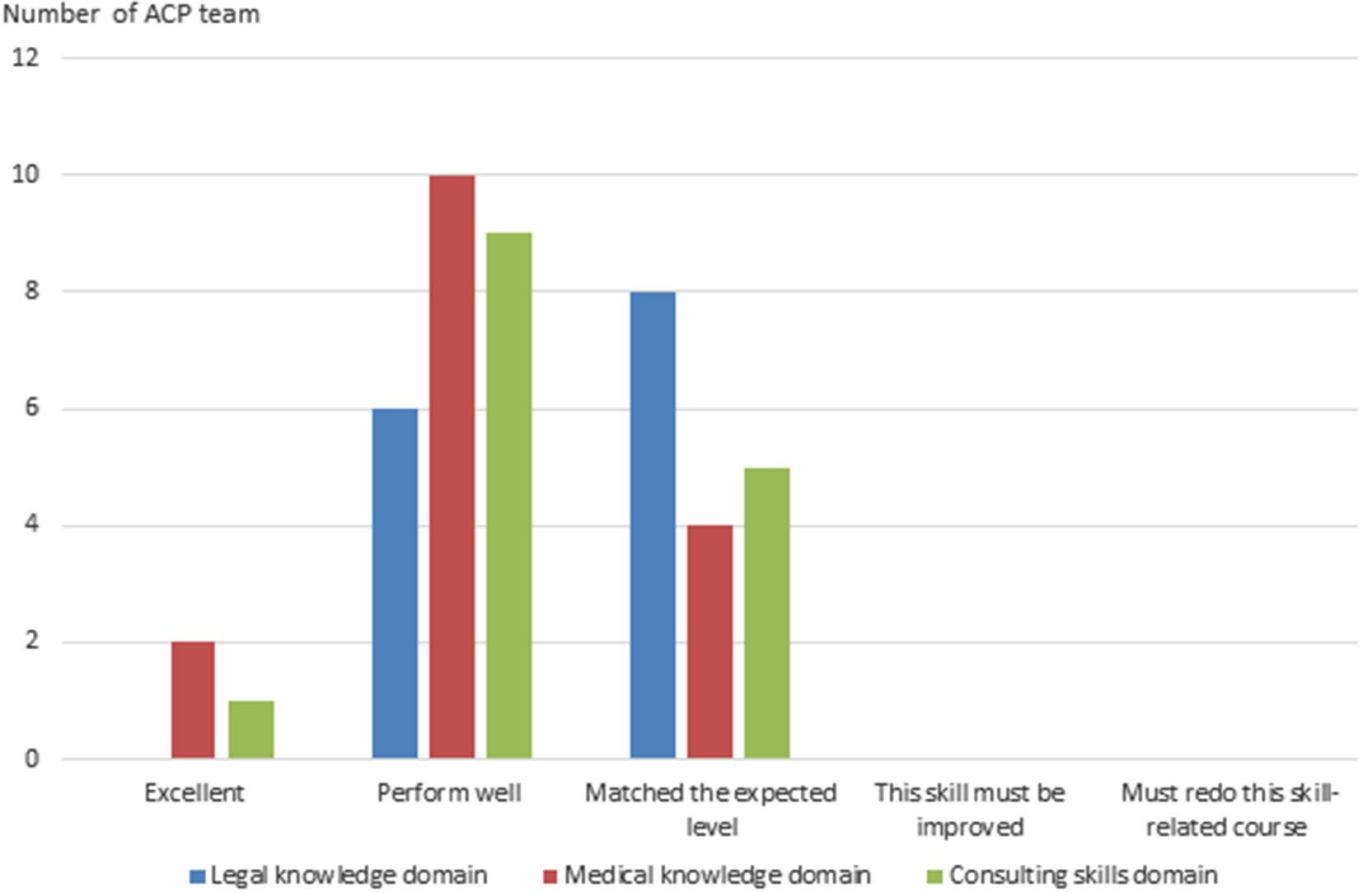
Score reflecting the 15 domains of the ACP performed by preceptors in their evaluation of each ACP team

Figure 1 reveals the score of the checklists reflecting the 15 domains of the ACP performed by preceptors in their evaluation of each ACP team. Most of the domains were completely or partially accomplished by the participants. However, some of the domains (Q6, Q7, and Q15) showed more negative results. Figure 2 shows the general performance of all ACP teams in legal knowledge, medical knowledge, and consulting skill. Most of the ACP teams were qualified for all standards.

Supplementary Fig. 1 reveals the score of the checklists reflecting the 11 domains of ACP conducted by SPs for each ACP team. For most of the domains, SPs gave a favorable or partially favorable response. However, some of the domains (Q6 and Q11) showed more negative results. After the ACP-OSCE exam, most SPs indicated that they would be willing to have the participants as their ACP professionals in real-world ACP.

**Table 1:** Demographic Characteristics of the Participants

Characteristics		Total (*n* = 38) N
		N
Male		18
Average age (year)		37.9
Occupation		
	Physician	15
	Nurse	15
	Social worker	5
	Psychologist	3
Region of hospital in Taiwan		
	North	15
	Central	11
	South	9
	East	3
ACP experience by profession (Mean cases)		
	Physician	54
	Nurse	66
	Social worker	31
	Psychologist	11
ACP experience by region of hospital (Mean cases)		
	North	64
	Central	30
	South	192
	East	90

The units of average age use mean age

**Table 2:** Average score before and after the exam of participants

		Before OSCE	After OSCE	P
Communication skills		3.47	3.84	< 0.05
Medical expertise		3.61	4.08	< 0.05
Legal knowledge		3.45	3.89	< 0.05
Empathetic response		3.58	3.87	0.07
Problem solving skills		3.47	3.97	< 0.05

**Table 3:** Average score before and after the exam by different occupations

		Before OSCE	After OSCE	P
Physician				
	Communication skills	3.60	3.93	0.055
	Medical expertise	4.07	4.33	0.164
	Legal knowledge	3.80	3.93	0.334
	Empathetic response	3.67	3.73	0.751
	Problem solving skills	3.71	4.14	0.054
Nurse				
	Communication skills	3.47	3.80	0.096
	Medical expertise	3.53	4.13	< 0.05
	Legal knowledge	3.40	4.00	< 0.05
	Empathetic response	3.60	4.13	0.056
	Problem solving skills	3.47	4.07	< 0.05
Social Workers and Psychologists				
	Communication skills	3.25	3.75	0.104
	Medical expertise	2.88	3.50	0.095
	Legal knowledge	2.88	3.63	< 0.05
	Empathetic response	3.38	3.63	0.563
	Problem solving skills	3.13	3.50	0.285

### Results of qualitative analysis

In our analysis, most of the participants mentioned that ACP has great benefits to the society and the people. However, some people also raised the difficulties of ACP implementation, such as legal or communication issues. However, most of the participants agree with the teaching method of using OSCE to ACP.

In accordance with participants’ responses collected, five distinct themes emerged:

#### Encourage students to sharpen their professional skills

Some participants responded that they want to improve their professional abilities, such as knowledge of law, how to familiar with clinical condition judgment, or how to counsel with dementia patients:

As a medical professional, I need more legal knowledge and more experience to protect myself from lawsuits. (from number 1)

For patients with dementia, I have some difficulties in determining whether the patient with adequate decision capacity. I think it is more challenging to judge the autonomous state of consciousness of patients with dementia, so more training is needed. (from number 4 )

#### Understand the importance of doctor-patient communication

In the questionnaire survey, most of the participants believed that communication is the most important bridge between doctors and patients in ACP process:

Through this OSCE program, I understand what patients care about and what they worry about. There is a big difference between a patient’s perspective and a doctor’s perspective, so communication is important. (from number 5)

Furthermore, some participants mentioned that the impact on communication may be related to public perception and cultural background:

Many people’s awareness has not been updated, and they often confuse DNR and euthanasia with AD. In addition, it seems that some areas have the culture to avoid discussing death, especially in areas with more aging populations such as rural area. (from number 8)

#### ACP brings the greatest benefits to the society

Many participants had feedback that ACP can make people have autonomy and guarantee the right to a good death. In society, it can help building awareness of death, breaking death taboos, promoting life education, and reducing ineffective medical treatment.

I think ACP can kindly guide citizens to fully express their expectations for end-stage medical treatment, and to know what they will face in advance, so that patients can respect themselves more and also reduce the pressure on family members. (from number 12)

#### Difficulties in ACP implementation

Many participants reported that ACP is expensive and not cost-effective. Some participants also reported that some medical staff resisted ACP and didn’t want to receive educational activities of ACP:

I think the difficulty of implementation of ACP lies in the lack of publicity by the government, enough reimbursements for consultations, and the resistance of medical staff to accept the concept of ACP. (from number 20)

Furthermore, some participants reported that there are some disadvantags which make it even more difficult to implement ACP.

When implementing ACP, it is difficult for the elderly with hearing impairement to be consulted, and some vulnerable members of the family are also easily ignored. There is often not enough time for everyone in the family to speak. (from number 13)

#### Perspectives of ACP-OSCE

Most of the participants agree with the teaching mode of OSCE. Because it is different from the previous teaching mode in the classroom, it can further enhance the understanding of ACP.

Using the OSCE model is challenging and rewarding, and I can see my own shortcomings. And I can learn more by exchanging experience with other hospital ACP teams. (from number 21)

OSCE is very helpful to my practical experience in the actual ACP consultations. (from number 31)

However, a small number of participants reported that OSCE would be difficult.

Participating in OSCE is a bit stressful and difficult, and it is difficult to convince other members of our ACP team to participate. (from number 5)

But most of the feedback was supportive. Some participants expressed the hope that it can be extended to all hospitals to learn ACP in the way of OSCE. There are also participants who think that even those healthcare professionals who already have ACP qualifications can still participate, because they can still improve their skills.

I hope this teaching is open to hospitals that do not participate in OSCE but have executive ACP teams to learn together. (from number 20)

I have been doing ACP for a while, but this OSCE can make me more aware of my shortcomings. (from number 34)

## Discussion

Communication skills and interpersonal relationships are the cornerstones of ACP. However, these skills are hard to evaluate, and OSCE provides an opportunity for better evaluation and education of ACP. To the best of our knowledge, this is the first study to explore the outcome of OSCE among qualified healthcare professionals in ACP education. Communication skills, medical expertise, legal knowledge, and problem-solving skills significantly improved among 38 healthcare professionals after OSCE implementation.

Overall, 4 of the 5 domains of performance improved after OSCE educational program implementation. Empathy was the only domain which did not show any improvement. Concerning the potential influence of a small sample size on observed differences, we employed Gpower software to calculate the statistical power for empathy, yielding a value of 0.67. However, for other dimensions such as Communication Skills and Medical Expertise, the power exceeds 0.8, specifically measuring at 0.94. We hypothesize that the observed variations may stem from inherent cultural disparities or differences in clinical aptitude. For example, participants exhibited favorable responses to empathy assessments before the OSCE, yet the growth in empathy was less pronounced after the OSCE. Besides, a previous study has shown that empathy is a basic core ability in medical education and also a cornerstone in high-quality health care [32]. Surprisingly, empathy is not easy to be established and some studies even found empathy declined during medical training, suggesting that the nature of empathy is unique, especially among healthcare professionals. Colliver et al. discovered that tools for evaluation of empathy, such as self-evaluation of medical students, may not truly represent meaningful results during clinical practice [33], and this may explain why empathy was not significantly improved in our study. In a recent systematic review, empathy could be improved by the following: (1) Sitting (versus standing) during the interview; (2) Detecting patients’ non-verbal cues of emotion; (3) Recognizing and responding to opportunities for compassion; (4) Non-verbal communication of caring (e.g., eye contact); and (5) Verbal statements of acknowledgement, validation, and support [34]. We believe that these skills can be evaluated by OSCE and could be implemented in OSCE checklists for the purpose of better evaluation of empathy.

Different outcomes were discovered among physicians and nurses. For physicians, the average score of the 5 domains of ACP skills increased without significance. For nurses, the average score of medical expertise, legal knowledge, and consultation problem-solving skills were significantly increased. In a previous study, Larson et al. found that there were many different viewpoints among physicians and nurses, including how collaboration and joint decision-making are valued, the definition of what constitutes adequate and appropriate interprofessional communication, the quality of nurse-physician interactions, and the understanding of respective areas of responsibility, as well as patient goals [35]. These differences could explain, at least in part, the discrepancies in our findings that were found among the various healthcare professionals in this study. Guner et al. found that nurses used a variety of methods that improve communication to a greater extent compared with physicians. Compared with physicians, nurses’ awareness of health literacy is higher and they are already better at incorporating health literacy-sensitive items into their practices [36]. We also found that before ACP-OSCE, the average score of physicians was higher than nurses, psychologists and social workers. This could be explained by that the role of physician existed in daily practice when discussing palliative care and end-of-life treatment with patients and family even before implementation of Patient Right to Autonomy Act, however other healthcare professionals had seldom chance to take part in ACP previously. This was also the reason why physicians felt less improved after ACP-OSCE program. With respect to ACP, physicians still play an important role, and thus future study is warranted to explore factors affecting successful ACP education for physicians.

Besides, it is very interesting that physicians felt no improvement in legal knowledge but nurses, social workers, and psychologists did. It is possible that legal knowledge is part of physician’s training in the past but other healthcare professionals seldom had similar training. However, from preceptors’ point of view, legal knowledge was still a relative weak spot among all ACP teams. Therefore, legal knowledge appeared to be a weak spot during ACP based on the preceptors’ evaluation. In the United States of America, 49–76% of ACP are completed with the participation of lawyers, but 6–7% of ACP are completed with physicians [37–39]. Hooper et al. discovered that lawyers have better legal knowledge and understand clients’ socioeconomic backgrounds and plans better than physicians [19]. A previous study showed that there is a significant disjunction between legal standards and physicians’ awareness of those standards [40]. For end-of-life law, research has shown that legal knowledge levels vary somewhat across professions, and legal knowledge gaps have been observed in all professional groups [41]. The results of our study also reflect the lack of legal knowledge of healthcare professionals. Although qualified ACP professionals need to receive legal education, our results suggest that their legal knowledge may be insufficient. Legal education programs should be enhanced for ACP professionals.

Most participants in our study felt improved in knowledge and skill of ACP after the program, and one characteristic of our ACP-OSCE program is debriefing after ACP. Debriefing is known to be the most important aspect of medical education [42]. Previous studies shown that debriefing could increase mutual understanding among preceptors and students and improve the outcome after educational process or workshop [43–45]. However, debriefing seldom occurs after ACP as we discovered in Taiwan. Our study supports that debriefing could be helpful for ACP healthcare professionals after ACP.

From the open-ended feedback and thematic analysis, we understand that how different stakeholders think and discuss ACP and the concept of death. Increased diversity in patient populations and healthcare personnel creates cross-cultural misunderstandings, leading to medical errors, lack of trust, and adherence to treatment [46, 47]. Medical staff therefore need to acquire more knowledge to improve cultural competence [48]. Therefore, we think our ACP-OSCE program provides such an understanding environment. In addition, most participants agreed to using OSCE in teaching, but some participants report that OSCE will be stressful and difficult. Early research has shown that more complex OSCE will have a more stressful response [49]. Therefore, it may be more suitable to start with simple OSCE design.

This study has some limitations study. First, the number of participants was limited, which may have caused sampling bias. However, although the number of participants was limited, we enrolled participants from each region/county of Taiwan, and we believe that the composition of participants is representative of Taiwan’s ACP professionals. Second, the evaluation of outcome is subjective, and thus it may be affected by an individual’s opinions, experience, personality, and so on. However, we also included the evaluations of preceptors and SPs in order to overcome this potential limitation. Further study is needed to develop a better, more objective method of evaluating the outcome of OSCE during ACP education.

## Conclusion

This is the first national ACP-OSCE program in Asia. The ACP-OSCE program helped ACP professionals to improve the efficiency of their counseling skills and enhanced their competency. We believe that our ACP-OSCE program could be applied in different countries to improve the ACP process, and to further increase patients’ overall quality of life.

### Electronic supplementary material

Below is the link to the electronic supplementary material.

Supplementary Material 1

## Data Availability

The datasets generated and/or analysed during the current study are not publicly available due to the anonymity of the participants but are available from the corresponding author on reasonable request.

## Abbreviations

ACP: Advance Care Planning
OSCE: Objective Structured Clinical Examination
SP: Standardized Patient
AD: Advance Decision
DNR: Do-Not-Resuscitate
DNH: Do-Not-Hospitalise
HCA: Health Care Agent
