# Supplementary material for: Developing an innovative national ACP-OSCE program in Taiwan: a mixed method study
Source: BMC Med Educ. 2024 Mar 23;24:333. doi: 10.1186/s12909-024-05294-5 (PMC10960391; doi:10.1186/s12909-024-05294-5)
Supplement: Supplementary file 1 — Supplementary Material 1 [file 12909_2024_5294_MOESM1_ESM.docx]

Supplementary Figure 1. Score of the checklists reflecting the 11 domains of ACP conducted by SPs for each ACP team

Supplementary Data 1. Scenarios of ACP and instructions for participants, preceptors, and standardized patients

1. Billboards

**Stop** **1**

Mr. Wang is 70years old and healthy person

1. Guideline for ACP healthcare professionals

⬛Background:

Mr. Zi Wang is a 70-year-old healthy person and has retired from a shoe factory. Because of his experience caring for his elderly mother, who had terminal cancer, he was very keen to find out more information about the “Patient Right to Autonomy Act”. Mrs. Wang, his wife, is not well educated and has worked as a housewife at home for many years. She has no understanding of the “Patient Right to Autonomy Act”. Mr. Wang has two children; the eldest son is the vice president of a technology company. Because of the requirement of “Patient Right to Autonomy Act” Mr. Wang’s son has been forced to participate in advance care planning by Mr. Wang.

Today, Mr. Wang has come to the hospital with his wife and son to arrange ACP.

⬛Topics of examination:

- Inform the participant about the rights and obligations of the law using teamwork
- Explain the options of advance decision (AD) and answer the questions appropriately
- Explain the specific clinical conditions in the “Patient Right to Autonomy Act”
- Explain the problems that participants and family may encounter when AD is executed
- Explain the role of the health care agent (HCA)
- Deal with emotions and special situations in consultation

⬛Test time: 40 minutes

1. Checklist

⬛Examination items:

1. The explanation of the disease
2. Communication skill
3. Health education

⬛Test time: 40 minutes

| Scoring items:(15 items). | Assessment of the consulting team | | | |
| --- | --- | --- | --- | --- |
| How completely did the team accomplish the following items? | Didn't do it | Partly done | Completely Done | note |
| 1. The host correctly introduces himself and the team and explains the consultative task |  |  |  |  |
| 1. Have the patient introduce himself and his family and confirm his or her identity |  |  |  |  |
| 1. Explain that the patient has the right to know, choose, and decide in accordance with the law |  |  |  |  |
| 1. Explain the specific clinical conditions that should be met for termination, removal, or non-implementation of life-sustaining treatment or artificial nutrition and fluid feeding. |  |  |  |  |
| 1. Explain the format of the advance decision and its statutory procedures |  |  |  |  |
| 1. Explain the procedure for amending and withdrawing the advance decision |  |  |  |  |
| 1. Explain the right of the health care agent (HCA) and the provisions for termination and dismissal of HCA |  |  |  |  |
| 1. Be able to communicate clearly in the language used by the willing person and family |  |  |  |  |
| 1. Explain hospice-palliative care in detail |  |  |  |  |
| 1. Show respect to the willing person and the family |  |  |  |  |
| 1. Assist the patient to complete and sign the AD |  |  |  |  |
| 1. Non-linguistic communication skill |  |  |  |  |
| 1. Counseling techniques: Guide the patient and family members to express needs |  |  |  |  |
| 1. To be clear and reasonable about the feelings and intentions of the patient, and to encourage the patient to express himself/herself. |  |  |  |  |
| 1. Describe the problems that may arise in the implementation and execution of AD in the future |  |  |  |  |

Overall performance (Legal knowledge domain).

| 5 Excellent performance Good enough to serve as a model for all consulting teams | 4. Performed well Good enough to serve as a model for young consulting teams | 3. Matched the expected level | 2. This skill must be improved | 1. Must redo t this skill-related course |
| --- | --- | --- | --- | --- |
|  |  |  |  |  |

Overall performance (Medical knowledge domain).

| 5 Excellent performance Good enough to serve as a model for all consulting teams | 4. Perform well Good enough to serve as a model for young consulting teams | 3. Matched the expected level | 2. This skill must be improved | 1. Must redo this skill-related course |
| --- | --- | --- | --- | --- |
|  |  |  |  |  |

Overall performance (Consulting skills domain).

| 5 Excellent performance Good enough to serve as a model for all consulting teams | 4. Perform well Good enough to serve as a model for young consulting teams | 3. Matched the expected level | 2. This skill must be improved | 1. Must redo this skill-related course |
| --- | --- | --- | --- | --- |
|  |  |  |  |  |

Any comments: Please try to provide any valuable advice; words are precious.

______________________________________________________________

Clinical preceptor (signature).

1. Guidance for preceptors

⬛The purpose of this examination is:

□ medical history inquiries □ physical examination□ interpretation and clinical treatment of the condition

⬛ Medical communication and health education □ individual skill operations

⬛Task for preceptors

1. The purpose of this examination is to pass the minimal standards for the ACP Counseling Team, not to identify the merits.
2. The key assessment items of this examination (Critical Decision point) are communication skills, medical knowledge, and dealing with difficult issues on the spot. Please pay special attention to these points.
3. The performance of the general ACP counseling team is expected as normal.[What does this mean?] (May be determined by the results of the examination or by the consensus of experts).

⬛Scenario: in an ACP center in hospital.

⬛Basic data of standardized patient: patient(70 years old), patient’s spouse (65 years old), patient’s son (40 years old).

⬛Starting Posture of standardized patient: Sitting Position.

⬛Summary of illness:

1. Main case
2. Situation:

Mr. Zi Wang, a 70-year-old healthy person has retired from a shoe factory. Because of his experience of caring for his elderly mother, who had terminal cancer, he has become very keen to find information about the “Patient Right to Autonomy Act”. Mrs. Wang, his wife, is not well educated and has worked for many years as a housewife at home. She has no understanding of the “Patient Right to Autonomy Act”. Mr. Wang has two children: the eldest son is the vice president of a technology company. Because of the requirement of the “Patient Right to Autonomy Act” Mr. Wang’s son has been forced to participate in advance care planning by Mr. Wang.

Today, Mr. Wang has come to the hospital with his wife and son to arrange ACP.

Mr. Wang has already received Pre-ACP counseling and has some knowledge of the law.

Emotions: the patient is firm, the wife is confused and panicky, the son is anxious and angry.

1. Chief complaint: For ACP
2. The ACP consultation team should complete the task.

1. Self-introduction and opening remarks

2. Confirm the legality of the participants.

3. Describe the rights and obligations of the patient in the “Patient Right to Autonomy Act”.

4. Describe the five specific clinical conditions.

5. Explain life-sustaining treatment.

6. Explain artificial nutrition and fluid feeding.

7. Explain the possible scenario at the time of execution of advance decision.

8. Answer questions from the patient and the family.

9. Deal with the feelings and emotions of the patient and the family

10. Explain the definition of health care agents.

C. Teaching assessment

1. Assess whether the consulting team's interpretation of ACP and the “Patient Right to Autonomy Act” is detailed and can calm the family's emotions.
2. Assess whether the consulting team explains the spirit and content of ACP.
3. Assess whether the consulting team can handle ACP consulting in an appropriate manner and with empathy.

If the ACP team has experience and progresses quickly, they can be evaluated at the next level:

1. Assess whether the consulting team can explain the relevant end-of-life options

2. Assess whether the consulting team can explain possible challenges in future implementation of advance decision

3. Notice the emotions and difficulties of the patient and family members and appropriately refer them to relevant resources for assistance (e.g., social worker, psychologist).

⬛Rating description:

1. The host appropriately introduces himself/herself and the team and explains the consultative mission. (Consulting skill domain).

- Fully completed: Provide a complete description of your full name and job title, as well as the team member’s name and job title, and explain the consultative assignment
- Partly completed: Host introduces himself/herself and the team and explains the tasks or processes, but only does one of them.
- Not done: No introduction of the host and the team, and no explanation of the consultation tasks and processes

1. Let the patient and the family introduce themselves and confirm their identity. (Legal knowledge domain).

- Fully completed: Invite the patient and family members to introduce themselves and their relationships, and confirm whether there are second-degree relatives of the patient who may wish to participate in the consultation.
- Not done: No invitation to the patient and family members to introduce themselves and their relationships

1. Explain that the patient has the right to know, choose and decide in accordance with “Patient Right to Autonomy Act”. (Legal knowledge low).

- Fully completed: Explain the relevant rights to the patient and the family, including the right to know, choose, and decide
- Partly completed: Explain the rights to the patient and the family, but not fully state the three rights to know, choose, and decide
- Not done: No explanation of the relevant rights to the patient and the family

1. When rated "Not done" for this score item, this indicates that the Consulting Team has neglected to provide important instructions related to the “Patient Right to Autonomy Act” to the patient and the family. Please give feedback to the consulting team to correct this oversight. Explain the specific clinical conditions to be met for termination, removal or non-implementation of life-sustaining treatment or artificial nutrition and fluid feeding. (Feedback key) (Medical knowledge domain).

- Fully completed: Details of life-sustaining treatment, artificial nutrition, and fluid feeding should be explained, including intubation, electric shock, CPR, ECMO, hemodialysis, antibiotics, blood transfusion, nasal gastric tube, percutaneous endoscopic gastrostomy, total parental nutrition, etc. And details of the definition of the five clinical conditions should be explained.
- Partially completed: There are instructions for life-sustaining treatment, artificial nutrition, and fluid feeding, but not all treatments listed above are described. Definitions of the five clinical conditions are given.
- Not done: No detailed explanation of life-sustaining treatment, artificial nutrition, and fluid feeding, or the five clinical conditions. (Feedback key)

Consulting team, if rated "Not done" for this score item, please invite the team for self-assessment at the time of feedback and recommend that the consulting team redo the advance care planning course.

1. Explain the format of the advance decision and its legal procedures (Legal knowledge domain).

- Fully completed: Explain the contents and options of the advance decision, as well as the subsequent signing, upload, inquiry, witness/notarization processes, and methods
- Partially completed: There are instructions on the contents and options of the advance decision, but no instructions on the subsequent signing, upload, inquiry, witness/notarization processes and methods.
- Not done: There is no explanation of content and options of advance decision, as well as subsequent signing, uploading, querying, witnessing/notarization processes and methods.

1. Explain the procedure for modification and withdrawal of the advance decision (Legal knowledge domain).

- Fully completed: There is a reference to the detailed procedure for advance decision that can be changed or withdrawn.
- Partially completed: There is a reference to advance decisions that can be changed or withdrawn without specifying the procedures for change or withdrawal.
- Not done: There is no mention at all that the advance decision can be changed or withdrawn.

1. Explain the rights of the health care agent and the requirements for termination and dismissal of health care agent (Feedback key) (Legal knowledge domain).

- Fully completed: There are explanations of the meaning, rights, requirements for termination, and dismissal of the health care agent.
- Partially completed: There is a description of the significance of the health care agent, but the relevant rights, termination, and dismissal process are only partially stated.
- Not done: Completely no explanation of the significance of health care agent; the patient is only asked whether he/she wishes to appoint a health care agent. (Feedback key)

Health care agents are important in the design of this Act and are involved in the options in advance decision, so it is important for the consulting team to explain the relevant regulations and to understand the attitude of the patient toward the health care agent.

1. Be able to communicate clearly in the language of the patient and the family. (Communication skills domain).

- Fully completed: Use the common language of the patient and the family, and confirm the degree of understanding between the patient and the family.
- Partly completed: The common language of the patient and the family can be used, but the degree of understanding between the patient and the family is not confirmed.
- Not done: Use of basic medical terms only and the degree of understanding between the patient and the family is not confirmed.

1. There are instructions on the treatment of palliative care

(Medical knowledge domain).

- Fully completed: Provide instructions on how palliative care works, such as pain management, after the advance decision has been executed. The function of consultation with the palliative care team before execution of advance decision is also explained.
- Partially completed: There are instructions for relevant palliative care, such as pain treatment, after the implementation of advance decision. However, the function of consultation with the palliative care team before execution of advance decision is not explained
- Not done: No explanation of the relevant palliative care treatment or the function of consultation with the palliative care team before execution of advance decision.

1. Respect for the patient and the family. (Consulting skills domain).

- Fully completed: to convey verbally or non-verbally the content and requests made to the patient or family member expressing considerate feelings
- Partially completed: Only one verbal or non-verbal expression was given
- Not done: Offensive language or behavior was used.

1. Assist the patient to complete and sign the advance decision (Feedback key) (Legal knowledge domain).

- Fully completed: Guide the patient and family members through the full contents of the advance decision and explain the meaning of the relevant options and how to complete the document.
- Partially completed: Explain to the patient and family members the contents of the advance decision, but the meaning of the relevant options is not explained and the patient is not shown how to fill in the document.
- Not done: There is no mention of any of the above. (Feedback key)

After the completion of advance care planning, it is still recommended that the consulting team guide the patient and the family through the contents of the advance decision, so it is advisable to prompt the consulting team to think in this direction.

1. Non-verbal techniques (Consulting skill domain).

- Fully completed: non-verbal techniques such as eye contact, reasonable physical contact, provision of paper, and timely pauses in interactions with the patient and family members can be used to achieve calming emotions and guide discussions.
- Partly completed: Maintenance of eye contact during the discussion, but physical or other nonverbal skills are not used.
- Not done: Rare eye contact with the patient and family members during communication (especially when explaining bad news or when family members are expressing important opinions).

1. Interview promoting techniques: Guide the patient and family members to demonstrate their needs (Feedback key) (Consulting skills domain).

- Fully completed: be able to use an open question in communication appropriately, guide the patient and family members to express needs, attitudes, values; after the patient or family members give an answer, ask whether there are other issues that still need to be discussed.
- Partly completed: An open question is used without further discussion, or the patient or family member is only asked if they have any questions without identifying possible needs.
- Not done: Only closed or countered questions (such as "You don't have any idea, do you?", " You're all right, aren't you? ") are used. The consulting team seem keen to end ACP. (Feedback key)

If the consulting team is rated "Not done" for this score item, please invite the team for self-assessment at the time of feedback and recommend that the consulting team redo the advance care planning course.

1. Be understanding and empathetic of the patient’s feelings person and encourage him/her to express themselves (Consulting skills domain)

- Fully completed: Respond to the emotions and feelings of the patient or family member using the same empathetic response technique (e.g., "You must be worried?" “I think it must be very difficult for the family to face…”, "You must be very hopeful that your husband doesn't leave you" are used more than twice during ACP.
- Partly completed: Only one empathetic response is used in the meeting.
- Not done: No empathetic response at all.

1. Discussion of problems that may arise when executing AD in the future

(Medical knowledge domain).

- Fully completed: Describe possible scenarios, steps, and related medical care during the execution of the AD in the future, and ask the patient or family member if they are concerned about this.
- Partly completed: Describe the possible scenarios, steps, and related medical care during the execution of the AD in the future, but the patient or family member is not asked if they are concerned about this.
- Not done: There is no discussion of possible scenarios, executional steps, and related medical procedures for the execution of the AD in the future. V. SP Guidelines (Scripts).

Description of the exam

⬛Topic: ACP

⬛Task:

1. Exam: Follow the SP’s guide to the script, consult with the consulting team during ACP, ask questions at the right time, and answer questions from the consulting team.

⬛Situation and starting position: Sitting position

⬛Emotions: the patient is firm, the wife is confused and panicky, the son is anxious and angry

⬛Manpower and items: 1 patient (Mr. Wang), 1 female standard family member (Mrs. Wang), 1 male standard family member (the son).

⬛Test time: 40 minutes

Principles of Responses to the Consulting Team

Actively ask questions, passively accept inquiries, and answer questions if the consulting team uses open-ended questions

Summary of the story

1. Main case

Situation:

Mr. Zi Wang is a 70-year-old healthy man who has retired from a shoe factory. Because of his experience of caring for his elderly mother, who has terminal cancer, he is very keen to find out more information about the “Patient Right to Autonomy Act”. Mrs. Wang, his wife, is not well educated and has worked for many years as a housewife at home. She has no understanding of the “Patient Right to Autonomy Act”. Mr. Wang has two children, and the eldest son is the vice president of a technology company. Because of the requirement of the “Patient Right to Autonomy Act” Mr. Wang’s son has been forced to participate in advance care planning by Mr. Wang.

Today, Mr. Wang has come to the hospital with his wife and son to arrange ACP.

Mr. Wang has already received Pre-ACP counseling and has some knowledge of the law.

Emotions: the patient is firm, the wife is confused and panicky, the son is anxious and angry.

1. The ACP consultation team should complete the following tasks:

1. Self-introduction and opening remarks

2. Confirm the legality of the participants.

3. Describe the rights and obligations of the patient in the “Patient Right to Autonomy Act”.

4. Describe the five specific clinical conditions

5. Explain life-sustaining treatment

6. Explain artificial nutrition and fluid feeding

7. Explain the possible scenarios at the time of execution of advance decision

8. Answer questions from the patient and family

9. Deal with the feelings and emotions of the patient and the family

10. Explain the definition of health care agents

1. Dialogue (Most ACP teams should proceed in most situations to at least the second stage, and for experienced teams, the SP can progress to the third or fourth stage as the situation progresses).

| Stage | Role | Dialogue | Remark |
| --- | --- | --- | --- |
| The first stage  (Opening stage with introduction) | Patient | I don't want anything. Don't save me. Let me pass on peacefully. |  |
|  |  | This is my wife, and this is my son. |  |
|  |  | What are the five specific clinical conditions? How can these conditions be so complex? |  |
|  |  | All these treatment, I don't want them, I don't want them at all. |  |
|  |  | Is signing this advance decision euthanasia? If I'm terminally ill, euthanasia is best! |  |
|  | Wife | Doctor, my husband called me, and I came and asked him what he was doing, and he couldn't tell me. What the hell is this? |  |
|  |  | If he doesn't receive any treatment, isn't it hopeless? |  |
|  | Son | Doctor, can you hurry up? I'm going to have a meeting later! |  |
|  |  | What, this conversation costs three thousand dollars? Isn't it free? |  |
| The second stage  (Explanation and description stage). | Patient | Life-sustaining treatment, I don't want it. I don't want it at all. |  |
|  |  | Nasal gastric tube, no! But a little intravenous fluid...I will consider it. |  |
|  |  | I've signed a DNR order before, and it didn’t cause me that trouble! |  |
|  |  | Health care agents? If it is necessary, I’ll find my wife, who knows me best. |  |
|  |  | What form do you want me to fill out? |  |
|  | Wife | If I sign this, is that giving up any kind of treatment? Without doing anything? |  |
|  |  | Will my husband starve to death without a nasal gastric tube? |  |
|  |  | What is a health care agent? I don't want to be……There's a lot of pressure…… |  |
|  |  | What if my husband changes his mind? Can advance decision be altered later? |  |
| The third stage  (Emotional Conflict Stage) | Son | Father! You called me here to listen to this nonsense? What do you mean you don’t want intubation and treatment? Please don't listen to the doctor - they're scaring you! |  |
|  |  | Illness needs treatment, and I will help my father to decide. We do not need you to intervene. |  |
|  |  | Well, dad, since you don't want any invasive life-sustaining treatment, there’s antibiotics, IV, blood transfusions…..they are not invasive and you should try them! |  |
|  | Wife | Old man, if you insist on doing this…. I'm the only one left in the world… I only have you. If you're not with me, I don't know what to do...(Crying). |  |
|  | Patient | Yes, my love, I have lived so long, and have such a good wife, and two successful children. I am satisfied... |  |
|  |  | Son, don't do this. These people are here to help us. You take care of yourself. I don't want to become your burden in the future. |  |
|  |  | Well, since my son insists on it, I still want to try antibiotics, IV, and blood transfusions, okay? |  |
| The fourth stage  (End-of-life options and problem resolution stage) | Patient | I'd also like to sign an organ donation form, okay? |  |
|  |  | Could my family cremate my body when I die and scatter the ashes at sea. Is that okay? |  |
|  |  | When I take my last breath, I don't want to be in the hospital. I want to be at home... How do you do that? |  |
|  |  | Okay, sign here… |  |
|  |  | Thank you. Thank you very much. Now I am finally satisfied. |  |

Scoring Sheet of SP

⬛Test topics: ACP and AD

⬛Test time: 40 minutes

⬛Test items:

| project | Degree of consent | | | |
| --- | --- | --- | --- | --- |
|  | Not  agree | Partially agree | Agree | very much agree |
| 1. I think the team really cares about us, and considers what is best for us | 0 | 1 | 2 | 3 |
| 2. After the team's interpretation, I am clearer about our rights and obligations under the Patient Right to Autonomy Act | 0 | 1 | 2 | 3 |
| 3. After the team helped me, I knew exactly what the five clinical conditions were. | 0 | 1 | 2 | 3 |
| 4. After the team explained, I was well aware of what life-sustaining treatment and artificial nutrition were. | 0 | 1 | 2 | 3 |
| 5. After the team explained, I was well aware of what a health care agent was. | 0 | 1 | 2 | 3 |
| 6. I think the team understands we're going to have a lot of pressure to sign this. | 0 | 1 | 2 | 3 |
| 7. The team is clear about the AD signature process and its legal effects. | 0 | 1 | 2 | 3 |
| 8. The team was attentive to whether we understood everything they said. | 0 | 1 | 2 | 3 |
| 9. The team made us feel like we didn't need to be afraid to ask the questions we wanted to ask. | 0 | 1 | 2 | 3 |
| 10. I think the team has noticed our emotions and used empathetic responses. | 0 | 1 | 2 | 3 |
| 11. I think the team was very clear about the end-of-life options. | 0 | 1 | 2 | 3 |

In terms of the overall performance of this team this time, if other friends and family would like to arrange ACP, which of the following statements do you most agree with?

□I'd love to. If other friends and family want to arrange ACP, I would recommend this particular team to them.

□I'd be happy to have this team in charge if other friends and family would like to arrange ACP.

□Yes, but it doesn't matter if another team does it instead.

□I would not like this team to arrange the ACP for my friend, but if there's really no other team that can do it, that would be OK.

□I'd suggest that my friend change to another hospital and find another team; otherwise don’t do it.

Any comments: Please try to provide your valuable advice; any words are precious

______________________________________________________________________

SP (Signatures).
